# First Reported Cases of Biomechanically Adaptive Bone Modeling in Non-Avian Dinosaurs

**DOI:** 10.1371/journal.pone.0131131

**Published:** 2015-07-08

**Authors:** Jorge Cubo, Holly Woodward, Ewan Wolff, John R. Horner

**Affiliations:** 1 Sorbonne Universités, UPMC Univ Paris 06, UMR 7193, Institut des Sciences de la Terre Paris (iSTeP), 4 Place Jussieu, BC19, F-75005, Paris, France; 2 CNRS, UMR 7193, Institut des Sciences de la Terre Paris (iSTeP), F-75005, Paris, France; 3 Montana State University, Museum of the Rockies, 600 West Kagy Boulevard, Bozeman, Montana, 59717, United States of America; 4 Department of Anatomy and Cell Biology, Oklahoma State University Center for Health Sciences, 1111 W. 17th St., Tulsa, OK, 74107, United States of America; 5 Small Animal Internal Medicine, Purdue University College of Veterinary Medicine, 625 Harrison Street, West Lafayette, IN, 47907, United States of America; Raymond M. Alf Museum of Paleontology, UNITED STATES

## Abstract

Predator confrontation or predator evasion frequently produces bone fractures in potential prey in the wild. Although there are reports of healed bone injuries and pathologies in non-avian dinosaurs, no previously published instances of biomechanically adaptive bone modeling exist. Two tibiae from an ontogenetic sample of fifty specimens of the herbivorous dinosaur *Maiasaura peeblesorum* (Ornithopoda: Hadrosaurinae) exhibit exostoses. We show that these outgrowths are cases of biomechanically adaptive periosteal bone modeling resulting from overstrain on the tibia after a fibula fracture. Histological and biomechanical results are congruent with predictions derived from this hypothesis. Histologically, the outgrowths are constituted by radial fibrolamellar periosteal bone tissue formed at very high growth rates, as expected in a process of rapid strain equilibration response. These outgrowths show greater compactness at the periphery, where tensile and compressive biomechanical constraints are higher. Moreover, these outgrowths increase the maximum bending strength in the direction of the stresses derived from locomotion. They are located on the antero-lateral side of the tibia, as expected in a presumably bipedal one year old individual, and in the posterior position of the tibia, as expected in a presumably quadrupedal individual at least four years of age. These results reinforce myological evidence suggesting that *Maiasaura* underwent an ontogenetic shift from the primitive ornithischian bipedal condition when young to a derived quadrupedal posture when older.

## Introduction

Intensive paleontological fieldwork over the last three decades has produced a rich collection of non-avian dinosaur fossils permitting detailed ontogenetic descriptions and paleobiological estimations of life history traits using bone histology (e.g., *Maiasaura* [1], *Tyrannosaurus* [2], *Allosaurus* [3]). These collections also allow analyses on the incidence of healed skeletal injuries, or bone abnormalities. Moderate to high incidences of healed skeletal injuries have been reported in natural populations of extant species: e.g., 64% (n = 61) in the Virginia opossum [4]; 36% (n = 118) in gibbons [5]; 15% (n = 308) in African viverrids [6]. We have found two cases of exostoses (4%; n = 50) in the tibiae belonging to an ontogenetic sample of *Maiasaura peeblesorum*. This study is aimed at testing hypotheses on the proximal causation of these bone outgrowths.

Bone periostitis is a common bone abnormality easily recognized by the expansion of diaphyseal contours (outgrowths) and involving an alteration in bone surface texture [7]. Such features are the outcome of three groups of aetiologies: trauma, local infection, and collateral effects of other diseases including neoplasms and metabolic diseases [8]. Our comparisons with modern vertebrates suggest that the reported exostoses in the tibiae of *Maiasaura* are the outcome of trauma (fibula fracture). Moreover, the topological position of these outgrowths is interpreted as evidence for an ontogenetic shift from a bipedal to a quadrupedal posture in *Maiasaura*.

## Material and Methods

Over more than thirty years, the Museum of the Rockies (MOR; Bozeman, MT) has prepared disarticulated *Maiasaura peeblesorum* fossils collected from a rich, monodominant bonebed in the Campanian sediments of the Two Medicine Formation [9]. Fifty tibiae from that bonebed were used in a population histology analysis, representing individuals from one year of age through skeletal maturity (Repository: Museum of the Rockies, Montana State University, 600 West Kagy Boulevard, Bozeman, Montana 59717 USA). The minimum number of individuals, based on the number of right tibiae, is 32. If each tibia represents a distinct individual, then the maximum number sampled is 50. Two tibiae (MOR 005-T9 and MOR 005-T42) exhibited exostoses. No permits were required for the described study because the fossils were collected on land owned by the Museum of the Rockies Inc.

Thin sections were prepared from 0.3 cm thick wafers of bone removed transversely from either side of the minimum diaphyseal circumference of *Maiasaura* tibiae. Thin section slides were processed using a Buehler Ecomet 4 variable speed grinder, using the following sequence of grit papers: 60, 180, 320, 600, and 800. Completed slides were analysed using a Nikon Optiphot-Pol polarizing microscope at either 10 X or 40 X total magnification, and photomicrographs were taken incrementally using a Nikon DS-Fi1 digital sight camera. The software package NIS-Elements BR 3.0 was used to create a single image from multiple photographs, so that composite images of the tibia thin sections have a mosaic appearance. Minimum age of *Maiasaura* individuals was determined by counting the number of annually deposited lines of arrested growth (see [10–12] for descriptions of skeletochronology methods). High resolution images of tibiae examined in this report can be downloaded from Morphobank (www.morphobank.org; project P2136).

Three types of analyses were performed on these sections: histological (identification of bone tissue types and qualitative comparisons of bone compactness), biomechanical (quantification of the maximum second moment of the area, proportional to the bending strength, and its orientation using BoneJ [13])), and paleopathological (analysis of possible explanatory aetiologies).

## Results and Discussion

**Fig 1:**
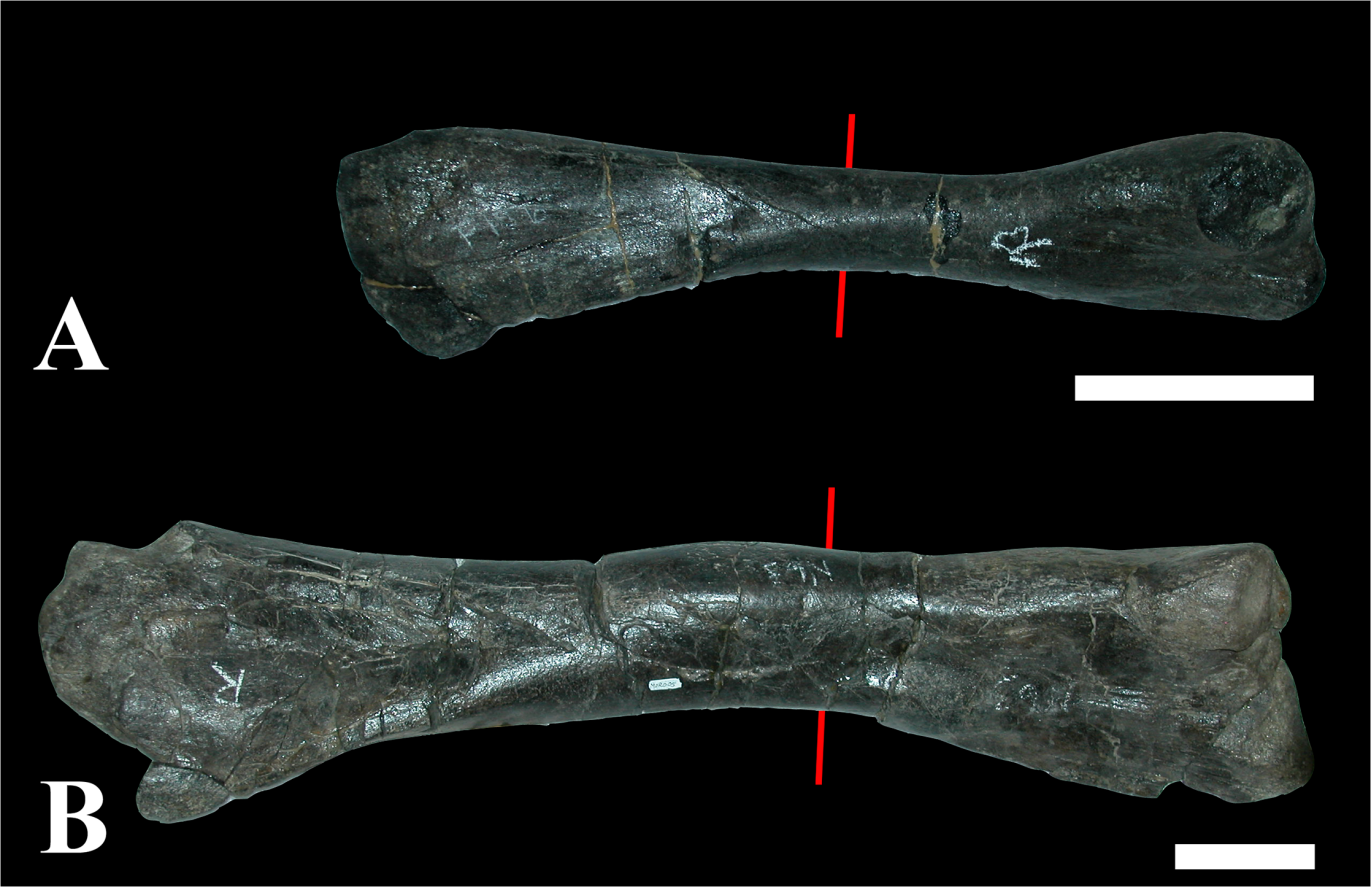
Overall views of the bones showing outgrowths. (A) Right tibiae of a one year old *Maiasaura* specimen and (B) a four year old specimen. The red lines indicate where the histological sample was taken. Proximal is to the left, distal to the right. Scale bar equals 10 cm.

**Fig 2:**
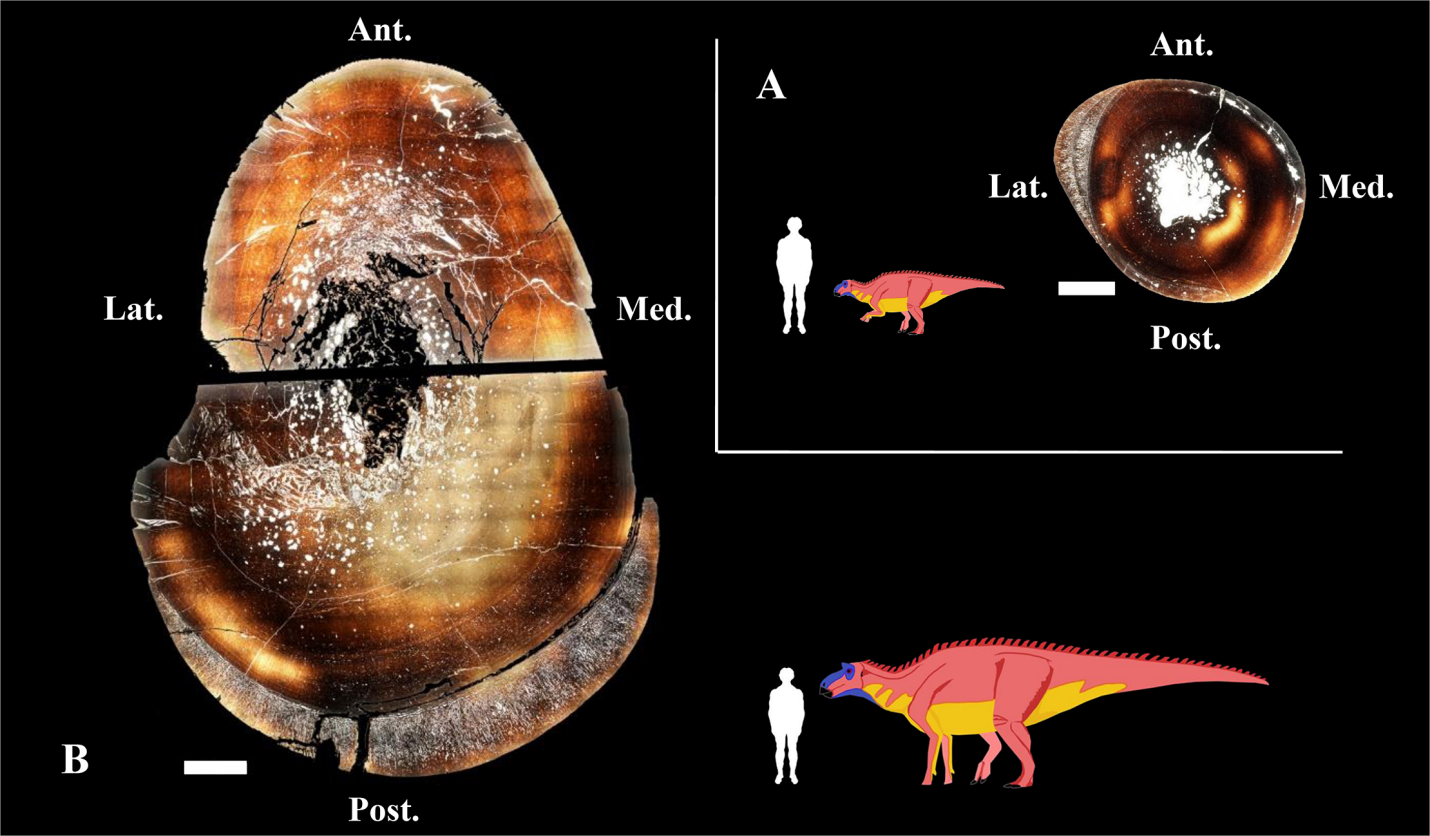
Bone cross-sections showing the general histological aspect of the outgrowths. (A) Right tibiae of a presumably bipedal *Maiasaura* specimen and (B) a presumably quadrupedal specimen. Scale bar for bone sections equals 1 cm. Abbreviations: Ant., anterior; Lat., lateral; Med., medial; Post., posterior.

The ontogenetic series of fifty tibiae of *Maiasaura peeblesorum* analyzed contains two tibiae (MOR 005-T9 and MOR 005-T42) exhibiting exostoses (Figs 1 and 2). On tibia MOR 005-T9, there is no visual indication of any lesion or outgrowth on the diaphysis (Fig 1). On MOR 005-T42, there is a distinct bulge in the bone, approximately 20 cm in length, but again the diaphyseal surface is smooth (Fig 1). Because many of the bones from this bonebed are tectonically distorted or deformed, the unusual appearance of this specimen went unnoticed until histologically sectioned.

Experimental work (osteotomy) simulating the effects of a fracture in a zeugopodial bone (the ulna) produces increased strains in the adjacent zeugopodial element (the radius), promoting bone modeling (bone formation occurs over primary bone) and/or remodeling (bone formation occurs on a surface previously resorbed by osteoclasts, intracortically, at the periosteum or at the endosteum) [14–16]. In adult sheep, ulnar osteotomy produces both intracortical remodeling and periosteal modeling in the radius [14, 15], whereas in still-growing young pigs ulnar osteotomy produces endosteal remodeling and periosteal “explosive” growth (modeling) in the radius [16]. Consistently, the exostoses found in the two *Maiasaura* tibiae are hypothesized to be cases of biomechanically adaptive periosteal bone modeling resulting from overstrain on the tibia after a fibula fracture.

### (a) Histological analyses

**Fig 3:**
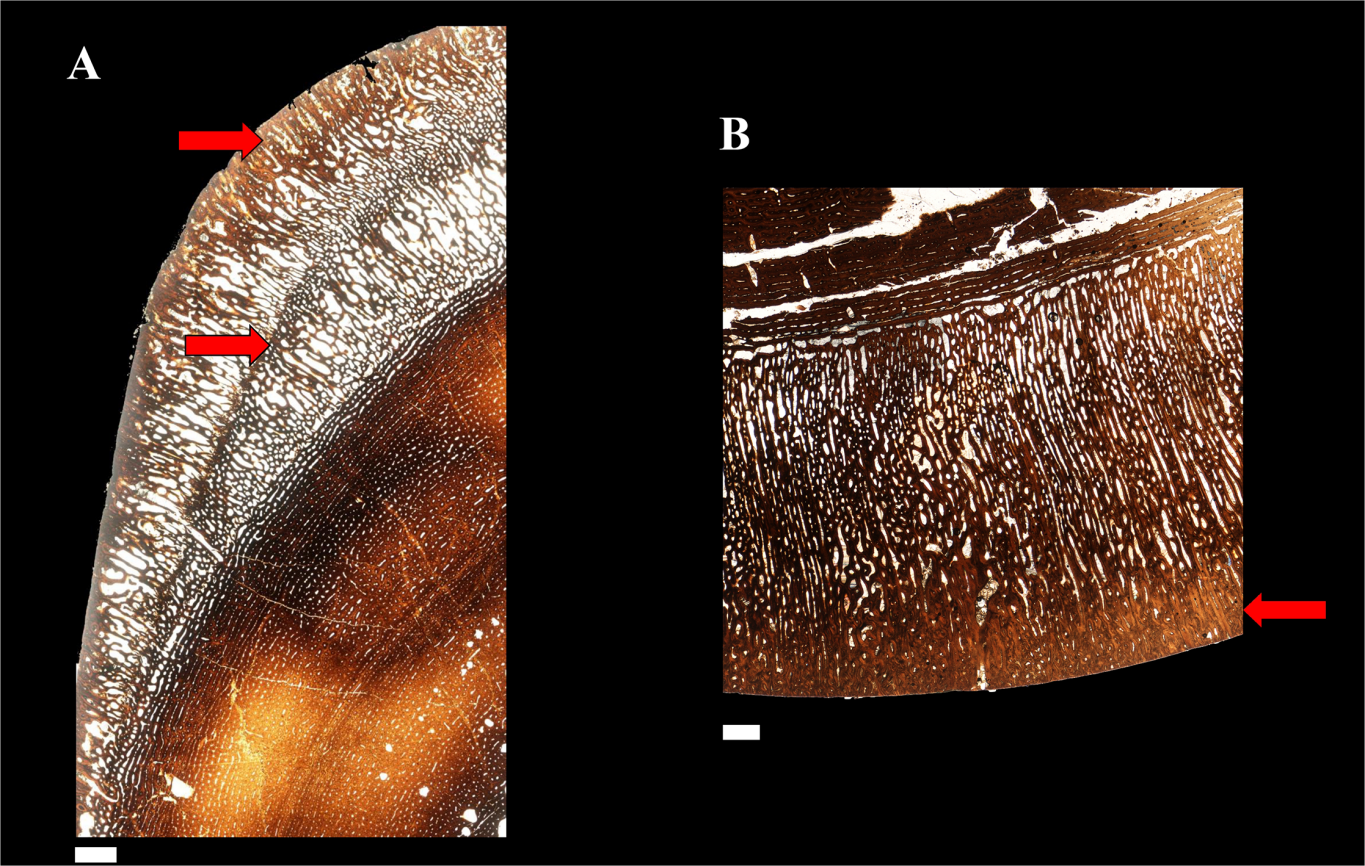
Detailed histological aspect of the outgrowths. Outgrowths are constituted by radial fibrolamellar periosteal bone tissue formed at very high bone growth rate [12, 13], to compensate the overstrain presumably produced by a fibula fracture. These outgrowths involve two pulses of periosteal growth in the one year old specimen (A) and a single pulse in the four years old specimen (B). Considering that the further from the neutral plane of bending, the higher the biomechanical constraints [14, 15], we expect higher compactness on the periphery of bone outgrowth to compensate for the increased constraints on the bone surface. Our observations support these predictions in both the first and second bursts of growth of the one year old specimen (A) and in the single burst of growth of the four years old specimen (B). Scale bar equals 1 mm.

The cortex of *Maisaura* tibia is mainly formed by laminar fibrolamellar bone tissue interrupted by lines of arrested growth (Figs 2 and 3). Fibula fracture may have produced instantaneous biomechanical overstrain on the adjacent tibia, necessitating rapid cortical compensation via directional outgrowths. So we expect to find a bone tissue formed at very high growth rates. The histological architecture of the *Maiasaura* tibial outgrowths consists of directional radial fibrolamellar periosteal bone tissue, involving two layers of periosteal growth in the small tibia (38.4 cm length) from a one year old specimen (MOR 005-T9) and a single layer in a larger tibia (90.5 cm length) from an immature individual at least four years of age (MOR 005-T42) (Fig 3). According to uniformitarianism, the same natural laws and rules (e.g. Amprino’s rule) that operate now have operated in the past. Amprino’s rule suggests a relationship between bone growth rates and bone tissue types [17]. Radial fibrolamellar bone tissue type is found in vertebrates under natural (up to 171 μm/day in the King Penguin [17]) and artificial (47 μm/day in the Chicken [18]) selection for very high bone growth rates. Thus the observation of radial fibrolamellar bone tissue in the outgrowths of *Maiasaura* suggests that they were formed at very high bone growth rates. Physical activity may stimulate rapid bone growth, particularly in young individuals. However, the discontinuity observed between the cortical bone and the outgrowths in both specimens (MOR 005-T9 and MOR 005-T42) suggests an abrupt change in mechanical constraints compatible with fibula fracture. Always in the context of uniformitarianism, the finding of rapidly formed radial fibrolamellar bone tissue in the young pig radius after ulnar osteotomy [16] is congruent with our hypothesis. The observed cortical response in the growing pigs [16] provides an appropriate modern analogue to the condition observed in the fossil tibiae. We compared the fibrolamellar complex of mammals and dinosaurs on the basis of their similarities in terms of histological structure, developmental mechanisms and function. However we do not know whether they are homologous (i.e., acquired by the last common ancestor of amniotes) or the outcome of convergent evolution (as occurs with many physiological and morphological traits linked to endothermy).

The second moment of the area (*I*), proportional to the bending strength of a long bone, is computed as:

$$I=\sum y^{2}\;z\;\delta y$$

where *z δy* is the cross-sectional area of a thin layer at a distance *y* from the neutral axis [19]. It is obvious that, all other things being equal, a compact layer of bone (i.e., with primary osteons filled by centripetal bone apposition) may produce a higher increase of the second moment of the area of a bone section (and a higher increase of the bending strength of the long bone), than a layer with low compactness (i.e., with primary osteons still unfilled by centripetal bone apposition). Moreover, we deduce from the same equation that the farther from the neutral axis, the higher the contribution of a thin layer to the second moment of the area of a bone section (and to the bending strength of the long bone [20]). Thus, considering that biomechanical constraints (i.e., tensile or compressive) acting on bone tissue during bending increase away from the neutral plane in a transverse section [19, 20], and that stresses should concentrate in the periphery of bone cortex, we expect to find greater compactness at the periphery of each outgrowth. Our histological results also agree with this prediction: both layers of localized periosteal growth observed in MOR 005-T9, and the single layer observed in MOR 005-T42, show higher bone compactness near the surface than deeper within the radial tissue of these outgrowths (Fig 3). In strong contrast, during normal long bone development, deep primary osteons are filled by centripetal bone apposition before the more recently formed, peripheral primary osteons (see for instance Fig 1E in [21]).

### (b) Biomechanical analyses

**Fig 4:**
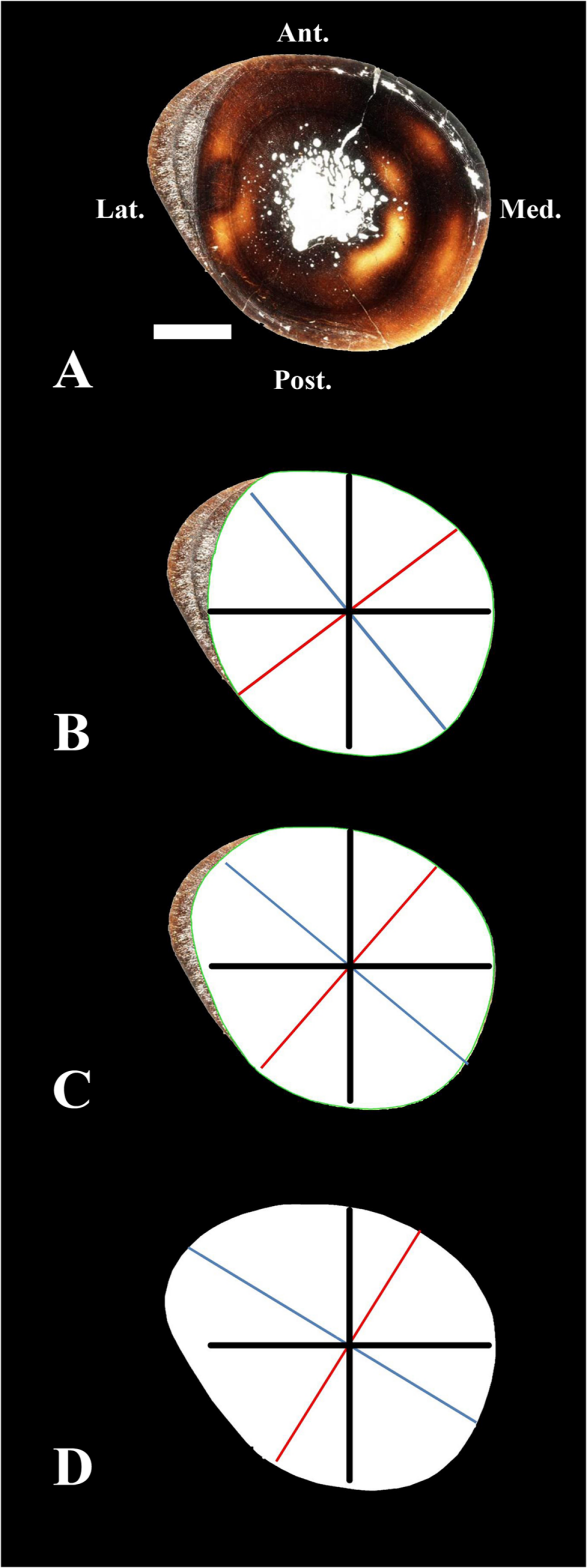
Biomechanical analysis of the outgrowths of the one year individual MOR 005-T9. Entire bone cross-section (A) and biomechanical analyses of bone areas (white surfaces) before fibula failure (B), after the first outgrowth subsequent to fibula failure (C), and after the second outgrowth (D). The black lines represent antero-posterior and latero-medial axes for reference. The blue line represents the maximum second moment of the area (Imax), which is proportional to the bending strength of the bone. The red line is the neutral plane. Before fibula failure, tibia Imax was oriented at 38° relative to the antero-posterior axis (B). After fibula failure, tibia Imax increased 13.8% towards the mediolateral axis (from 38° to 49°), as expected in a presumably bipedal, one year old specimen (C). The second burst of growth further increased tibia bending strength (34.6% relative to the situation before the trauma) towards the mediolateral axis (from 49° to 58°) (D). Scale bar equals 1 cm. Abbreviations: Ant., anterior; Lat., lateral; Med., medial; Post., posterior.

Because the tibia naturally experiences bending moments during locomotion, the exostoses observed in *Maiasaura* tibiae would increase the diaphyseal second moment of the area *(I*, proportional to the bending strength [22]). Experimental strain recordings in the sheep tibia show that, during peak loading, this bone withstands craniocaudal bending with maximal tensile strains located on the anterior surface, and maximal compressive strains located on the posterior surface [23]. Data on tibia deformation obtained using an *in vivo* optical approach in humans suggest that this bone mainly withstands craniocaudal bending, but also mediolateral bending and torsion [24]. Summarizing, it has been shown that tibia withstands craniocaudal bending in a quadruped (sheep [23]), whereas this bone withstands craniocaudal bending but also mediolateral bending in a biped (humans [24]). Myological evidence suggests that *Maiasaura* underwent an ontogenetic shift from a bipedal condition to a quadrupedal posture [25]. So we expect to find the outgrowths in a more or less medio-lateral axis in the tibia of the presumably bipedal, one year old, specimen MOR 005-T9, and in an antero-posterior axis in the tibia of the presumably quadrupedal, at least four years of age, individual MOR 005-T42. Our results are congruent with these predictions. Before fibula failure, tibia Imax was oriented at 38° relative to the antero-posterior axis in MOR 005-T9 (Fig 4B). After fibula failure, the first burst of localized periosteal growth in the tibia produced a 13.8% increase in the maximal second moment of area towards the medio-lateral axis (from 38° to 49°counterclockwise relative to the antero-posterior axis), as expected considering the strain measurements obtained in a bipedal tetrapod [24] (Fig 4C). The second outgrowth pulse produced a further Imax increase (of 34.6%), also towards the medio-lateral axis (from 49° to 58°) (Fig 4D). *Maiasaura* tibia MOR 005-T42 reveals a single outgrowth, increasing Imax by 39.6% but in the antero-posterior axis (as expected, considering the strain measurements obtained in a quadrupedal tetrapod [23]). This interpretation is based on empirical data obtained in two species, so it should be accepted with caution pending additional experimental data. Moreover, relationships between patterns of bone loading and sites of periosteal bone formation are complex [23, 26, 27]. Our interpretation relies on the classic assumption that functional loading promotes bone formation in regions that experience the highest strains [28, 29]. However, an increasing number of studies have found evidence for a different mechanism: functional loading stimulates bone formation at sites experiencing small strain magnitudes (e.g. [23, 26]). These last results are congruent with the load predictability hypothesis according to which bone curvature and elliptic cross-sectional shape with a minor axis aligned with the direction of bending may decrease bone strength but may increase load predictability by promoting a preferred bending direction [30]. Moreover, during sheep ontogeny, increased functional loading promotes periosteal modeling in proximal midshafts, whereas it induces cortical Haversian remodeling in distal midshafts [31]. Periosteal modeling decrease and intracortical Haversian remodeling increase with age [31]. In our view, all these hypotheses are not mutually exclusive: (i) Moderate functional loadings may stimulate bone formation at sites experiencing small strain magnitudes to increase load predictability, provided that a sufficient safety factor to withstand unexpected loads is preserved. (ii) Extreme functional loadings (e.g., following trauma) involving small safety factors and a risk of fracture may stimulate bone formation at sites experiencing the highest strains. Experimental data obtained in the sheep radius are congruent with our view and support our interpretation for the functional causation of *Maiasaura* outgrowths. Sheep radius shows bone curvature in the sagittal plane (see Fig 1 of [15]), and an elliptic cross-section with a major axis in the latero-medial direction (see Fig 2A of [15]), so that the preferred bending direction (determined by the plane of curvature and the direction of the minimum second moment of the area of the bone cross-section) is craniocaudal. Experimental strain recordings agree with this prediction and show that sheep radius withstands craniocaudal bending, with the concave caudal surface under longitudinal compression and the convex cranial surface under longitudinal tension [15]. After ulnar osteotomy, radius withstands higher functional loadings involving reduced safety factors. As expected, bone formation occurs at the caudal surface of the radius, where the highest strains were recorded [15]. Similarly, *Maiasaura* outgrowths may indicate the regions experiencing the highest strains after fibula fracture.

### (c) Paleopathology analyses

Describing modified bone structure as resulting from modeling requires that scenarios regarding disease be ruled out. As noted, in MOR 005-T9 and T42, there is a collar of fibro-lamellar bone lying beneath the periosteal layer of the tibia. This can be observed grossly as an area of smooth bowed thickened bone on the diaphysis (Fig 1). Although this style of bone forming beneath the periosteum is characteristic of a number of lesions, it readily becomes clear that there is no ‘good fit’ paleopathologic hypothesis. Perhaps the simplest lesion is a periosteal reactive lesion, which involves uplift of the periosteum and rapid deposition of benign bone [32]. Grossly, this can be marked either a smooth thickened area of bone, or the bone texture can appear irregularly emarginated and have a roughened texture. The appearance of periosteal reactive bone is not consistent. This is one type of lesion described previously in stegosaurs [8]. These lesions are unlikely to produce a uniform cuff of bone as they must by nature be localized to the insult. Unlike recent stegosaur work [33], there is no erosive endosteal component described histologically to suggest osteomyelitis in these specimens. Abscesses, although known to produce periosteal reactive bone growth in mammalian Brodie’s abscesses [34], are characterized by pus, which is a neutrophilic fluid not present in archosaurs. Instead, cordoned off bone infections are characterized by heterophil induced caseous fibriscesses [35] which should not present the same impetus for fibrolamellar bone deposition. The outward appearance of such lesions therefore will lack draining sinuses, but may appear wildly proliferative with an open cavity that would once have contained caseous necrotic debris. Bone neoplasia such as osteomas and osteosarcomas are described in avian wildlife [36], however these lesions are generally more focal, and in the case of osteosarcoma frequently involve severe alterations to bone density, underlying structure and integrity. Grossly, osteomas are button-like smooth lesions, sometimes having a mildly roughened texture but otherwise having very well delineated margins. Osteosarcomas can arise from the bone margin (parosteal osteosarcoma), or can be have more medullary involvement and tend to have a very expansile proliferative appearance.

Perhaps the most compelling similarity to a pathologic condition is avian osteopetrosis [37–41]. This skeletal lesion develops as a consequence of the avian leucosis virus, and differs from human osteopetrosis in that the human condition involves primarily cartilaginous and medullary resorption defects rather than periosteal bone deposition targeting [37]. On gross pathology, the bone may have a latticework collar of bone surrounding the diaphysis and may have very minimal density bone deposits proximal to the metaphysis. Previous papers have described this condition in archaeology era birds from early Britain [39], and alluded to its presence in dinosaurs [40, 41]. Noticeably absent from the two MOR *Maiasaura* specimens in this paper is the circumferential deposition of bone around the entire diaphysis that characterizes this condition. Additionally, both individuals lack evidence of the classic second phase of the disease in which the trabecular and endosteal bone becomes highly decreased in density [37].

In sum, the known pathologic scenarios fall short of explaining the bone histology observed in these two specimens. The authors grant that a scenario for a pathologic etiology for which there is no modern analog [42, 43] or a lesion for which examination of the complete skeleton would have yielded a different interpretation is always possible. However, the parsimonious argument for a biomechanical explanation (as supported by this paper) makes this possibility less likely given that the gross and histopathologic appearance of the bone does not suggest pathologic novelty.

## Conclusions

Paleopathology hypotheses (periosteal reactive lesions [8, 32, 33], osteomyelitis [34], fibriscesses [35], bone neoplasia [36], and avian osteopetrosis [37, 41]) fail to explain the presence of the observed exostoses in the tibiae of *Maiasaura*. In contrast, the hypothesis according to which these outgrowths are cases of biomechanically adaptive periosteal bone modeling after fibula fracture are strongly supported by histological and biomechanical data. The quantification of the frequency of (observed or inferred) bone fractures is important in paleontological population studies. Moreover, by documenting the differences in the compensating biomechanical responses in the tibiae between *Maiasaura* juveniles and sub-adults, our study independently supports the hypothesis that *Maiasaura* underwent an ontogenetic shift from bipedality to quadrupedality previously suggested by a myological analysis [25]. Evidence for ontogenetic postural change in turn suggests that immature dinosaurs exhibit ancestral character states (in the case of *Maiasaura*, bipedality), whereas derived character states (i.e., quadrupedality) did not develop until late-juvenile or sub-adult stages of growth, further implying the need to consider that small-bodied dinosaurs with unique combinations of shared and derived characteristics may in fact be immature morphs of derived taxa.
